# Triceps Surae Muscle Characteristics in Spastic Hemiparetic Stroke Survivors Treated with Botulinum Toxin Type A: Clinical Implications from Ultrasonographic Evaluation

**DOI:** 10.3390/toxins13120889

**Published:** 2021-12-12

**Authors:** Marco Battaglia, Lucia Cosenza, Lorenza Scotti, Michele Bertoni, Marco Polverelli, Alberto Loro, Andrea Santamato, Alessio Baricich

**Affiliations:** 1 Physical and Rehabilitation Medicine, Department of Health Sciences, Università del Piemonte Orientale, 28100 Novara, Italy; alessio.baricich@med.uniupo.it; 2 Physical and Rehabilitation Medicine, “Ospedale Maggiore della Carità” University Hospital, 28100 Novara, Italy; 3 Rehabilitation Unit, Department of Rehabilitation, “Santi Antonio e Biagio e Cesare Arrigo” National Hospital, 15121 Alessandria, Italy; luc.csnz@gmail.com (L.C.); mpolverelli@ospedale.al.it (M.P.); 4 Department of Translational Medicine, Università del Piemonte Orientale, 28100 Novara, Italy; lorenza.scotti@uniupo.it; 5 Physical Medicine and Rehabilitation, ASST Sette Laghi, 21100 Varese, Italy; michele.bertoni@asst-settelaghi.it; 6 Physical Medicine and Rehabilitation, Spasticity and Movement Disorder Unit, Policlinico Riuniti, Università di Foggia, 71122 Foggia, Italy; andrea.santamato@unifg.it

**Keywords:** muscle spasticity, spastic equinovarus foot, botulinum toxin, diagnostic imaging, ultrasonography, diagnostic techniques, rehabilitation, Provide data about the evolution of spastic muscles and the correlation of muscle fibroadipose degeneration with BoNT-A.

## Abstract

Equinovarus foot is one of the most commonly spasticity related conditions in stroke survivors, leading to an impaired gait and poor functional performances. Notably, spastic muscles undergo a dynamic evolution following typical pathophysiological patterns. Botulinum Neurotoxin Type A (BoNT-A) is the gold standard for focal spasticity treatment, and ultrasound (US) imaging is widely recommended to guide injections and monitor muscle evolution. The role of BoNT-A in influencing muscle fibroadipose degeneration is still unclear. In this study, we analyzed medial gastrocnemius (MG) and soleus (SOL) US characteristics (cross-sectional area, muscle thickness, pennation angle, and mean echo intensity) in 53 patients. MG and SOL alterations, compared to the unaffected side, depend on the spasticity only and not on the BoNT-A treatment. In functionally preserved patients (functional ambulation classification, FAC > 3; modified Ashworth scale, MAS ≤ 2), the ultrasonographic changes of MG compared to ipsilateral SOL observed in the paretic limb alone seems to be due to histological, anatomical, pathophysiological, and biomechanical differences between the two muscles. In subjects with poor walking capability and more severe spasticity, such ipsilateral difference was found in both calves. In conclusion, BoNT-A does not seem to influence muscle degeneration. Similar muscles undergo different evolution depending on the grade of walking deficit and spasticity.

## 1. Introduction

The first definition of spasticity was given by Lance in 1980 as a motor disorder characterized by a velocity-dependent increase in muscle tone with exaggerated tendon jerks, due to hyperexcitability of the stretch reflex, as a typical aspect of the upper motoneuron lesion [1].

Gracies and colleagues described the pathophysiology of spastic paresis, focusing on the motor control axis and its post-stroke evolution. The deficit of central nervous control causes a motor unit loss, leading to paresis, spasticity, overactivity, and, ultimately, disuse and immobilization complications. This generates a vicious circle in which spasticity and disuse feed off each other [2]. Thus, spastic muscle is a dynamic structure with complex evolution patterns that are still not completely clear.

In stroke survivors affected by spastic paresis, a common clinical find is spastic equinovarus foot (SEF) due to an increased tone of plantar-flexor muscles (PF). The main actor of plantar flexion is triceps surae muscle (TS), composed by medial and lateral gastrocnemii (MG and LG) and soleus (SOL). TS is in charge of 80% of torque in plantiflexion [3] and is among the most commonly affected muscles in case of spasticity [4].

Clinically, SEF may present as reduced ankle dorsiflexion and abnormal foot–ground interaction with impaired foot clearance, leading to augmented fall risk. Compensatory movements, such as ipsilateral hip circumduction or contralateral vaulting, performed to obtain a better foot clearance, can increase energy expenditure.

Botulinum Neurotoxin Type A (BoNT-A) demonstrated to be an effective and safe treatment for focal spasticity [5], with a low prevalence of complications [6]. For such reasons, BoNT-A injection is currently the gold standard for focal spasticity treatment [7,8].

The mechanism of action of BoNT-A consists of the inhibition of the pre-synaptic release of acetylcholine, thereby blocking neuromuscular transmission and inducing a transient chemodenervation of the injected muscle [9]. However, in order to maintain the clinical effect, BoNT-A injections should be performed every 4–6 months [10].

As previously pointed out, Gracies et al. described the evolution of the spastic muscle, reporting fibroadipose infiltration and contractile mass loss. Interestingly, due to its pharmacological mechanism of temporary denervation, the possible role of the chronic treatment with BoNT-A on spastic muscle evolution has been widely discussed [11].

Several studies quantitatively documented the grade of fibroadipose infiltration performing biopsies of BoNT-A injected muscles. In children with cerebral palsy (CP), adults with blepharospasm, an overactive bladder, or even healthy inoculated muscles, non univocal results were obtained [12,13].

Ultrasound guidance is highly recommended for BoNT-A injection in routine settings. In fact, there is a close relationship between injection accuracy and BoNT-A efficacy for both deep and superficial muscles [14].

Additionally, musculoskeletal ultrasound can be used as a good diagnostic tool for muscles, tendon, and joint disorders, which are generally assessed with a semi-quantitative approach. In this context, the US allows a safe and non-invasive follow-up of spastic muscles evolution over time, by measuring parameters, such as muscle thickness (MT, cm), fiber pennation angle (α, °), fascicle length (cm), cross-sectional area (CSA, cm^2^), and fibroadipose degeneration (measured with grey scale, MEI: mean echo intensity or MGV: mean grey value, scale 0–255) [15,16]. Those parameters are strongly related to muscle tension and its capacity to generate torque [17], and a higher echo intensity seems to determine a reduced response to BoNT-A [18]. Moreover, there is a significant correlation between increased MGV and interstitial infiltration of fibrous and fat tissue [19].

Based on these considerations, this study aims to assess changes in ultrasonographic muscle structure in stroke survivors, in relation to the focal spasticity treatment with BoNT-A.

The primary endpoint is the evaluation of muscular structural changes in MG and SOL in hemiparetic lower limb, in relation to the number of BoNT-A treatment cycles performed and the duration of the chronic treatment.

The secondary endpoint investigates the role of functional parameters and grade of spasticity in influencing the muscular evolution in spastic MG and SOL.

## 2. Results

Fifty-three subjects were enrolled in the period between December 2020 and June 2021. Table 1 shows the demographic characteristics of the study sample.

Results show a significantly impaired structure of paretic MG and SOL compared with the contralateral unaffected ones in terms of MT, CSA, pennation angle, and MGV, as shown in Table 2. In particular, this difference is statistically related to the post-stroke paresis alone, without any other apparent correlation, as seen in Table 3 and Table 4.

Notably, there is no significant correlation between BoNT-A injections and muscle structural alteration over time (Table 5). In particular, considering soleus ∆ MT, a p value of 0.0013 stands for a Spearman’s rank correlation coefficient (r) statistically different from 0. However, Spearman’s coefficient is considered significative when *r* < −0.6 or *r* > 0.6, as our value of 0.4314 makes the correlation not significant.

By comparing ipsilateral MG and SOL in the whole study sample, a worse value of MGV emerges in the gastrocnemii compared to solei, as seen in Table 6.

Stratifying the subjects according to functional walking parameters (functional ambulation classification, FAC) and the grade of spasticity measured with the modified Ashworth scale (MAS) results in Table 7 show an increased MGV in the paretic MG compared to ipsilateral SOL in patients with conserved walking function (FAC > 3) and lower spasticity level (MAS ≤ 2). On the other hand, in case of poor walking capability (FAC ≤ 3) and higher spastic hypertonia (MAS > 2), the same difference is detectable in the unaffected limb too.

## 3. Discussion

In our study population, we observed no relevant influence of BoNT-A in contributing to tissue degeneration in treated spastic muscles.

In subjects with preserved walking capability and lower spasticity grade, we detected an increased grade of fibroadipose infiltration in spastic MG compared to the ipsilateral SOL. Patients with impaired gait and severe spasticity presented these differences bilaterally.

It must be pointed out that current scientific literature is not univocal in defining the role of BoNT-A towards injected muscles degeneration.

BoNT-A provides a reversible pre-synaptic pharmacological denervation, which lasts 3 to 6 months [10], and it has been suggested that this chronic treatment may influence the spastic muscle evolution, leading to possible denervation-induced damages on treated targets.

In an animal model, Fortuna et al. investigated the effect of monthly injections of BoNT-A on unilateral hindlimbs muscles (rectus femoris, medial and lateral vasti) in healthy New Zeland white (NZW) rabbits. Results suggested how neuromuscular blockage induces severe muscle atrophy, weakness, and loss of contractile tissue even after a single injection. The deficit appeared to be directly related to the number of inoculation repetitions. Surprisingly, a considerable loss of strength was measured even in the contralateral limb only in the group with more treatment cycles performed. However, it was not possible to estabilish if such unexpected weakness was directly related to BoNT-A treatment or a result of disuse atrophy [20]. Interestingly, the same authors investigated the role of electrical stimulation (ES) exercise in a healthy NZW rabbit population treated with BoNT-A injections on unilateral rectus femoris muscle. By comparing the BoNT-A-only group and the BoNT-A + ES group, muscle biopsies showed how the program of ES exercise performed on both hindlimbs partially prevented the fibroadipose degeneration in injected muscles, and fully prevented contractile mass depletion in non-injected muscle [21].

In humans, muscle biopsies of BoNT-A-treated muscles showed variable outcomes. For example, Harris et al. reviewed muscle biopsies of patients with blepharospasm who were either treated or not treated with BoNT-A. They found no consistent longstanding alterations in muscle morphology in the BoNTA-treated group. Similarly, Haferkamp et al. showed no change in the ultrastructure of the detrusor muscle up to 11 months post-BoNT-A injection in overactive neurogenic bladder. On the other hand, Gough and colleagues have raised concern regarding the long-term effect of BoNT-A on muscle morphology in children with CP, revealing a reduced volume in the injected muscle and a coexistent hypertrophy of synergistic muscles [12]. However, several studies about children with CP describe an initial decline in muscle mass few months after the first treatment, followed by an almost complete recovery in a year time [22].

Furthermore, Schroeder et al. documented neurogenic atrophy in the injected MG in two healthy adults at 3, 6, 9, and 12 months post-BoNT-A [13].

Other studies suggest that muscle atrophy may be induced by pharmacological denervation with type I fiber loss and type II fiber predominance, significantly related to the number of cycles with BoNT-A [12]. Still, others ascribe the MG deficiency as not influenced by the number of treatment cycles in 12 months [23].

In our study sample, we observed a significant structural alteration of spastic MG and SOL, in which muscle bellies appear thinner, with higher fibroadipose degeneration and decreased fiber pennation angle, as expected in a stroke survivor paretic limb [24]. This implies an impaired contractility and consequent poor motor function [11,25].

In our population, the structural differences seem to be related to the spastic paresis. Other factors, such as sex, age, time elapsed stroke, number of treatment cycles, and duration of BoNT-A therapy, have no apparent correlation with muscle degeneration, according to our results.

A recent study evaluating the possible degenerative effect of BoNT-A on calf muscles in CP-affected children supports our statement thanks to an MRI examination of MG and SOL that corroborates the hypothesis of a non-significant role of BoNT-A in determining a meaningful fibroadipose infiltration [26].

Our findings strongly suggest that even a prolonged treatment with BoNT-A, characterized by several injection sessions per year (typically one every 3-6 months in our Centre), early followed by a cycle of intensive conventional neuromotor rehabilitation, does not influence muscle degeneration in a meaningful way.

Fonseca et al. described the therapeutic effectiveness of BoNT-A in association with the neuromotor rehabilitation treatment in children with CP; these two interventions have a synergic interaction which improves long-term functional outcomes [27].

In post-stroke patients, the combination of a standardized self-rehabilitation program following BoNT-A injections demonstrated to significantly potentiate the drug effect and improve lower limb function and gait performance [28].

For this reason, physiotherapy is widely implemented after BoNT-A injections and provides a longer-lasting therapeutic effect [5].

Further considerations could be made about ipsilateral rheological differences between MG and SOL. These muscles usually receive the same BoNT-A dose and are nearly always treated simultaneously.

Considering the whole study population, it emerges how both medial gastrocnemii undergo a more pronounced fibroadipose infiltration compared to corresponding solei, implying a differential decay even in the unaffected and normo-innervated limb.

Subgroup analysis clarified that, in patients with adequate walking capability (FAC > 3) and lower level of plantar flexors spasticity (MAS ≤ 2), ipsilateral degenerative difference is detectable in the paretic limb only. A similar observation can be found in Fortuna et al.’s animal model, where distinct but similar muscles (medial and lateral vasti) underwent different structural modification despite a comparable dose of BoNT-A and the same number of injections [20].

A hypothesis about the unequal grade of muscle degeneration involves a variable response of different muscles to BoNT-A injection. Several studies performed on animal models show how repeated inoculations lead to an increased expression of type I fibers and a concomitant depletion of type II fibers. However, the grade of muscle atrophy detected in children with CP treated with BoNT-A is lower than that observed in animal models [29].

This structural difference may be explained through several anatomical, functional, biomechanical, and histological differences between MG and SOL.

Firstly, subjects with preserved gait function tend to have a quite physiological physical activity in everyday life, which might help to maintain muscle characteristics. Differently, elderly patients with poor motility suffer from an early decay in the quality of contractile tissue [30], especially if laying in a stroke-inducted hypomobility condition. It has also been hypothesized that the deficit on the unaffected limb (confirmed in chronic stages) may depend on the disuse-inhibited central motor drive. Moreover, long-term limb disuse leads to lowered cortical excitability in the areas involved in controlling said body parts. However, an answer about progressive failure of voluntary activation in chronically disused nonparetic muscles may only be found with a longitudinal study [11].

Secondly, MG and SOL have a different composition in type I (mitochondrial rich, slow twitch, ST) and type II (myosin heavy chain rich, fast twitch, FT) fibers. In particular, the soleus is structured by almost 80% of type I fibers, versus 57% of the medial gastrocnemius [31]. This aspect implies a different metabolic pattern in as much as SOL carries out a mainly aerobic activity while MG performs predominantly anaerobic glycolysis [32].

An interesting aspect is a major depletion of “fast muscles”, such as MG, due to a prevalent loss of type II fibers [11], which are highly vulnerable to denervation. In a para-physiological process called age-related sarcopenia, the progressive loss of motor axons directed to type II fibers causes a collateral reinnervation by adjacent slow twitch fibers and a progressive histological switch to slow fibers. In stroke survivors, such mechanism seems to be reversed; Scherbakov et al. observed a prevalence of type II fibers with more reliance on anaerobic metabolism linked to a more severe gait impairment [33]. As described by Gracies et al., both situations often coexist in the same subject, bringing to the pathological re-distribution of muscle fibers and particular damage of “fast muscles”.

Furthermore, it must be pointed out that MG and SOL have different biomechanical roles in determining gait cycle phases.

MG is a biarticular muscle which works as plantar flexor and knee flexor. Its main activity is a concentric contraction that allows an active ankle flexion to maintain the stance phase and generate the limb propulsion. Due to type II fibers predominance, MG can perform faster contractions, mainly when associated with a knee extension, contributing to MG lengthening and consequent increased contractility. On the other hand, SOL is a monoarticular muscle and its length depends on the angle of plantar flexion alone. Its main role is to maintain the stance phase, controlling small pendular movements between the tibia and the astragalus, and slowing the tibial front drop during gait. This activity is performed with eccentric contractions that require a prevalence of type I slow fibers [3].

In stroke survivors with SEF, the decreased request of impulsive force and the general hypomobility may explain the increased depletion of type II fibers and MG-relative fibroadipose degeneration compared to SOL. In SEF gait, in fact, there is an abnormal foot–ground interaction: a “flat foot” or forefoot initial contact modifies the direction of ground reaction force, reversing the propulsion of PF. The MG, being the major player in propulsion generation, is, in this case, counterproductive and in biomechanical disadvantage. To this end, BoNT-A injections in PF could improve ankle dorsiflexion and allow a better foot–ground interaction.

Furthermore, a combined rehabilitative approach merging BoNT-A pharmacological treatment and physical exercise through a multidisciplinary team is widely recommended. Roche and colleagues measured superior gait performances and lower limb functional parameters in hemiplegic patients treated with BoNT-A and a self-rehabilitation program compared to BoNT-A alone [28]. Similar results occur in children affected by CP, in which the combination of conventional physical exercise, BoNT-A, and orthoses improves functional mobility in a synergic way [5,27]. Additionally, several adjuvant non-pharmacological treatments (e.g., taping, casting, orthoses, shock waves, ultrasounds, vibrations, and electrical stimulation) are available and commonly deployed in the post-injection phase. Picelli et al. reviewed the level of evidence of such techniques providing a panel of fruible alternatives. Unfortunately, a univocal administration protocol is still not defined [34].

The experimenters are aware of several limitations of this study.

Firstly, the sample size is relatively small; even if the study power is adequate, the generalizability of our results to the general stroke survivor population is not warranted.

Secondly, we analyzed a fairly heterogeneous population with a wide variability of age, grade of spasticity, functional parameters, and level of disability. Even though we did not observe a significant correlation of said variables with muscle tissue characteristics, these aspects may work as confounders, affecting the rheological properties.

Finally, this is a cross-sectional study. In order to throughly describe rehological characteristics of spastic muscles treated with BoNT-A, a prospective longitudinal design with follow-up ultrasonogrraphic evaluations will be taken in consideration.

## 4. Conclusions

Our findings confirm that BoNT-A is a safe treatment for SEF, that repeated injection cycles do not seem to induce fibroadipose infiltration in MG and SOL, and that muscle degeneration appears to be primarily related to the spastic muscle evolution.

The higher level of fibroadiposis observed in MG compared to ipsilateral SOL is bilateral in patients with poor walking performances (FAC ≤ 3) and higher spasticity level (MAS > 2). On the other hand, subjects with preserved functional performances present such difference only in the paretic limb.

The authors support post-injection rehabilitation programs, adjuvant treatments, and adequate physical activity which seem to have a preserving role on contractile tissue depletion, gait, and motricity [5,27,28,34].

Further studies are needed in order to better clarify these aspects in the general stroke survivors population. In addition, the implementation of elastography may be a complementary tool to qualitatively and quantitatively describe soft tissues structural properties in future researches.

## 5. Materials and Methods

This is a single-centered, cross-sectional, observational study set in the Physical Medicine and Rehabilitation Unit of “Ospedale Maggiore della Carità” University Hospital, Novara, Italy.

We enrolled participants among the stroke survivors affected by post-stroke spasticity (PSS) who were already addressed to our unit for the usual and periodic clinical re-evaluation and eventual BoNT-A treatment as an outpatient regime. The study participation was assessed only after clinical and instrumental detection of adequate criteria to perform the focal spasticity treatment with BoNT-A.

BoNT-A doses were chosen on the basis of the drug’s producer data sheet and on clinical evaluations, such as the grade of spasticity (measured with MAS), muscle volume, and the total number of muscles treated [35]. However, the ratio dose/muscle mass is not quantifiable, so the correlation between BoNT-A dose and US characteristics has not been investigated.

For study participation, all the patients gave their written informed consent structured according to the Declaration of Helsinki and approved by the local Ethics committee (CE register number 160/21) and the Competent Authority (Ospedale Maggiore della Carità University Hospital, Novara, Italy. Protocol 0016937/21, validated on 28 June 2021).

This study was registered on ClinicalTrials with the identifier NCT05097482.

The inclusion criteria were unilateral ischemic or hemorrhagic stroke (documented with clinical examination and neuroradiological findings), presence of spasticity at calf muscles (at least grade 1+ in the modified Ashworth Scale, MAS), and age greater than 18 years old. The exclusion criteria were inability to walk before stroke; presence of severe cognitive impairment; presence of other musculoskeletal, neurological, or cardiopulmonary comorbidities which could interfere with clinical findings; presence of skin lesions which could contraindicate BoNT-A treatment; and previous surgical myotendinous elongation, neurotomy, or rhizotomy.

Eligible patients for the study were assessed through ultrasound, which is already in use to guide BoNT-A inoculation.

We acquired images of the medial gastrocnemius (MG) and soleus (SOL). Images were processed through the software ImageJ (National Institutes of Health, USA) in order to measure the muscle thickness (MT, cm), the cross-sectional area (CSA, cm^2^), the pennation angle (α, °), and the mean gray value (MGV, range 0-255). MGV was assessed considering the whole muscle longitudinal section as region of interest (ROI). The gray scale spreads from 0 (black) to 255 (white).

Lateral gastrocnemius was not taken into consideration as it has a minor role in comparison to MG and SOL in determining plantar flexion due to a smaller muscle mass. Moreover, its biomechanical and histological characteristics are similar to MG [3].

Enrolled patients performed a single-session real-time B-mode ultrasonography using a linear multi-frequency transducer (scanning frequency 3–13 MHz, MyLab Six system, EsaoteSpA, Genoa, Italy).

The subject was in sitting position, with hip and knee flexed at 90°, the ankle in neutral position, and the feet leaning on a hard surface parallel to the sitting plane. For MG, the probe was placed at the junction between proximal and intermediary third of the calf in correspondence of the maximal CSA of the muscle. For SOL, the probe was placed in order to visualize the medial gastrocnemius myotendinous junction on the upper-left corner of the screen in longitudinal position.

We lightly placed the probe perpendicularly on the skin using water-soluble transonic gel. The same operator performed all measurements for every subject in order to prevent experimenter bias. Images were acquired in transversal and longitudinal sections on both affected and not-affected lower limbs [36]. We acquired three images for each section lifting, replacing the probe every time in order to prevent fortuitous artifacts. We took the average value among the three measurements of each variable.

**Figure 1:**
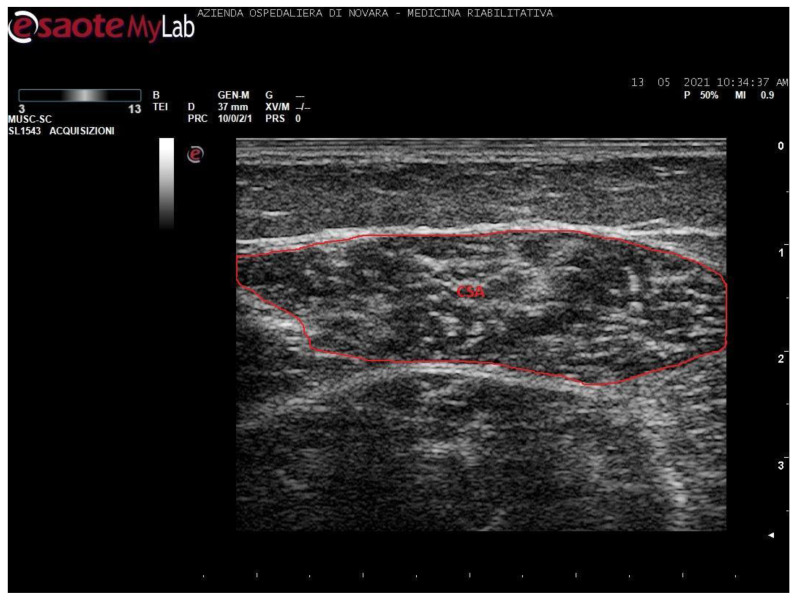
Ultrasound image of medial gastrocnemius in transversal section. In red: cross-sectional area (CSA).

**Figure 2:**
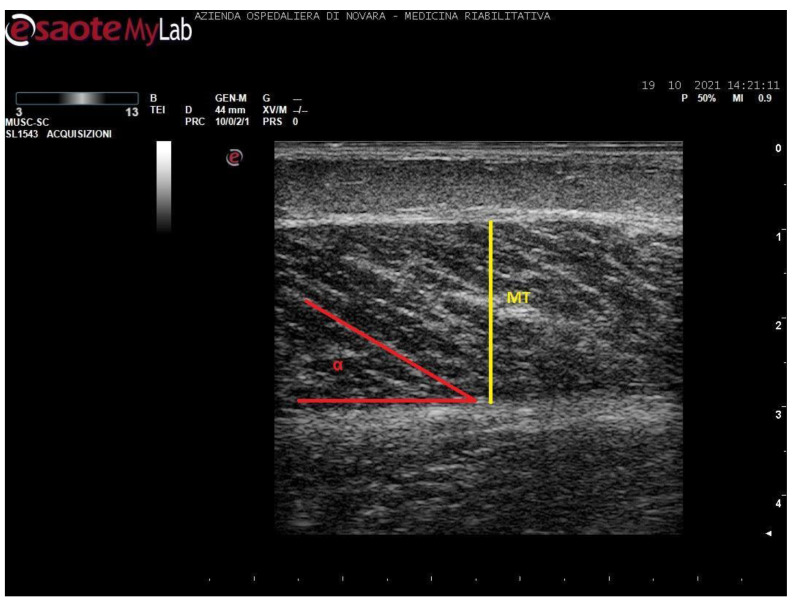
Ultrasound image of medial gastrocnemius in longitudinal section. In yellow: muscle thickness (MT), in red: pennation angle (α).

**Figure 3:**
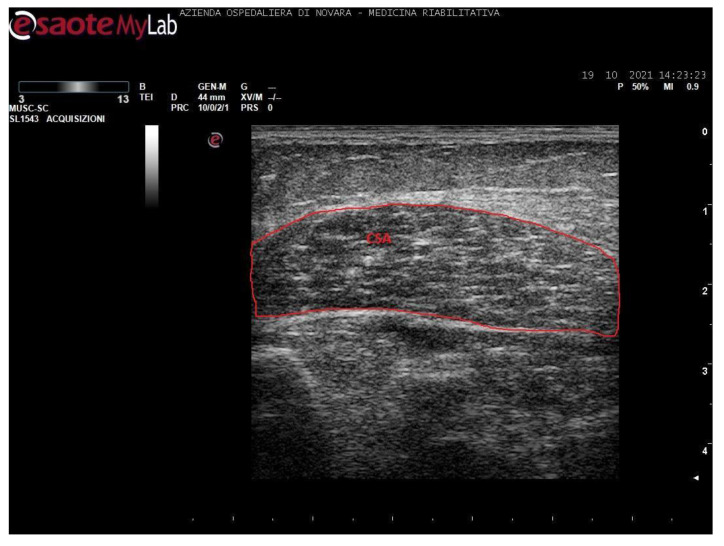
Ultrasound image of soleus in transversal section. In red: cross-sectional area (CSA).

**Figure 4:**
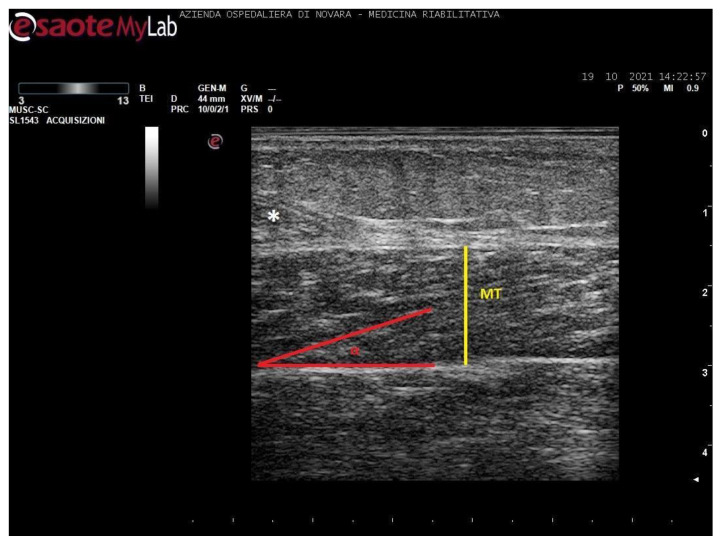
Ultrasound image of soleus in longitudinal section. In yellow: muscle thickness (MT), in red: pennation angle (α), *: myotendinous junction of medial gastrocnemius.

Measurements were taken as shown in Figure 1, Figure 2, Figure 3 and Figure 4.

Notably, soleus CSA presents several technical issues for its measurement. Firstly, in most cases, it is impossible to incorporate the entire area of such a wide muscle in the ultrasonographic frame. Secondly, the deep aponeurosis of SOL does not appear to be uniformly defined in transversal sections of long-standing degenerated spastic muscles, thus preventing an accurate profile definition. For these reasons, the authors preferred not to include such a biased value in this paper. The techinique of SOL CSA measurement was anyway reported in a healthy muscle in Figure 3 for illustrative purposes.

The functional ambulation classification (FAC) was assessed to measure gait capability [37].

Spasticity severity was measured with the modified Ashworth scale [38].

Data were collected in a single session (T0), without providing any different treatment from the common clinical practice.

Given a first type error of 0.05 and a power of 90%, a sample of 50 subjects was estimated to highlight a relative difference in the composition of the SOL of 25% [39], assuming a correlation between the measurements at within-patient equal to 0.4.

Descriptive statistics was calculated to summarize the characteristics of interest of the subjects included in the study: the categorical variables are reported as absolute frequencies and percentages while continuous variables as mean and standard deviation or median and first and third quartiles if not normally distributed. The normal distribution of data was assessed with the Shapiro–Wilks test. The paired t-test was applied to verify if the volume and composition of the MG and SOL differ between the hemiparetic and the healthy sides and between MG and SOL within sides. The latter comparison was performed for MGV stratifying subjects according to by FAC (≤3 vs. >3) and MAS (≤2 vs. >2) scores to assess if the differences were due to patients’ ambulation ability and grade of spasticity. Multivariate linear models for repeated measures were applied to estimate the difference in volume and composition between hemiparetic and healthy sides adjusted for other characteristics of the subjects. Paretic muscle ultrasonographic parameters variation (Δ), compared to the contralateral side, was determined by subtracting the values of the healthy limb to the affected limb ones, in order to obtain the mean difference. Finally, Spearman correlation coefficients were used to assess the correlation between volume and composition parameters and number of treatment’s cycles.

All tests were two-sided and the type I error was set to 0.05. Statistical analyses were performed using SAS version 9.4 (Cary, NC SAS Institute. Inc., Cary, NC, USA).

## Figures and Tables

**Table 1:** Clinical and demographic data of study population.

	*N* = 53
**Variable**	***N* (%)**
**Sex**	
Females	24 (45.28)
Males	29 (54.72)
**Hemiparetic side**	
Right	26 (49.06)
Left	27 (50.94)
	**median (Q1–Q3)**
**N treatment cycles**	6 (4–8)
**Time stroke—first treatment (years)**	1.05 (0.51–6.08)
**Time first treatment—acquisition (years)**	2.62 (1.54–3.93)
**Time stroke—acquisition (years)**	4.75 (2.45–10.33)
**Age at first dose—mean (SD)**	60.83 (10.90)
**Age at acquisition—mean (SD)**	63.75 (11.21)

**Table 2:** Structural ultrasonographic differences between medial gastrocnemius (MG) and soleus (SOL) of the paretic lower limb compared with the unaffected contralateral limb. SD: standard deviation, CSA: cross sectional area, MT: muscle thickness, α: pennation angle, MGV: mean gray value. Significance level: *p* < 0.05.

	Hemiparetic Limb	Unaffected Limb	Change	*p*-Value
	Mean (SD)	Mean (SD)	Mean (SD)	
Medial gastrocnemius				
CSA (cm^2^)	5.40 (1.16)	6.39 (1.25)	−1.00 (1.13)	<0.0001
MT (cm)	1.25 (0.25)	1.45 (0.26)	−0.20 (0.30)	<0.0001
α (°)	21.50 (5.96)	27.49 (5.14)	−5.99 (5.65)	<0.0001
MGV (0–255)	85.67 (21.87)	65.44 (18.54)	20.23 (19.68)	<0.0001
Soleus				
MT (cm)	1.52 (0.47)	1.70 (0.48)	−0.17 (0.43)	0.0035
α (°)	15.55 (4.49)	21.12 (5.8)	−5.57 (5.81)	<0.0001
MGV (0–255)	71.53 (15.93)	54.88 (14.82)	16.65 (14.54)	<0.0001

**Table 3:** Influence of demographic, clinical, and therapeutic factors on medial gastrocnemius structural changes between affected and unaffected limb. CSA: cross sectional area, MT: muscle thickness, α: pennation angle, MGV: mean gray value; β: mean value of variation of the dependant variable in relation to independent variable modification, SE: standard error. Significance level: *p* < 0.05.

	Medial Gastrocnemius							
	CSA		MT		α		MGV	
	β (SE)	*p*-Value	β (SE)	*p*-Value	β (SE)	*p*-Value	β (SE)	*p*-Value
Paretic vs. Unaffected Limb	−0.996 (0.155)	<0.0001	−0.204 (0.041)	<0.0001	−5.987 (0.776)	<0.0001	20.234 (2.703)	<0.0001
Age at First Treatment	−0.035 (0.014)	0.0148	−0.007 (0.003)	0.0132	0.000 (0.064)	0.9955	0.244 (0.236)	0.3071
Sex M vs. F	0.198 (0.297)	0.5082	0.023 (0.059)	0.7037	−1.119 (1.369)	0.4175	−4.078 (5.035)	0.4217
Time from Stroke	−0.014 (0.037)	0.7158	−0.003 (0.007)	0.6858	−0.086 (0.171)	0.6177	0.288 (0.634)	0.3843
N° Treatment Cycles	−0.059 (0.067)	0.3841	−0.016 (0.013)	0.2394	−0.365 (0.307)	0.2404	0.372 (1.13)	0.7432

**Table 4:** Influence of demographic, clinical, and therapeutic factors on soleus structural changes between affected and unaffect-ed limb. CSA: cross sectional area, MT: muscle thickness, α: pennation angle, MGV: mean gray value; β: mean value of variation of the dependant variable in relation to independent variable modification, SE: standard error. Significance level: *p* < 0.05.

	Soleus					
	MT		α		MGV	
	β (SE)	*p*-Value	β (SE)	*p*-Value	β (SE)	*p*-Value
Paretic vs. Unaffected Limb	−0.179 (0.059)	0.0035	−5.575 (0.798)	<0.0001	16.647 (1.997)	<0.0001
Age at First Treatment	0.002 (0.006)	0.7813	−0.001 (0.058)	0.9858	0.245 (0.174)	0.1658
Sex M vs. F	0.161 (0.124)	0.2008	−0.976 (1.228)	0.4303	−9.556 (3.669)	0.0120
Time from Stroke	−0.002 (0.016)	0.8864	0.181 (0.157)	0.2544	−0.693 (0.47)	0.1462
N° Treatment Cycles	0.015 (0.017)	0.3883	0.03 (0.169)	0.8610	0.296 (0.504)	0.5599

**Table 5:** Spearman’s rank correlation coefficient (r) between the number of treatment cycles and paretic muscle ultrasonographic parameters variation (∆). CSA: cross sectional area, MT: muscle thickness, α: pennation angle, MGV: mean gray value. Significance level: *r* < −0.6 or *r* > 0.6 and *p* < 0.05.

	N° Treatment Cycles
	*r* (*p*-Value)
Medial Gastrocnemius	
∆ CSA	0.0554 (0.6936)
∆ MT	−0.0257 (0.8551)
∆ α	−0.2107 (0.1299)
∆ MGV	0.1997 (0.1518)
Soleus	
∆ MT	0.4314 (0.0013)
∆ α	−0.0605 (0.6671)
∆ MGV	0.1734 (0.2143)

**Table 6:** MGV difference between ipsilateral MG and SOL on both affected and unaffected limb considering the whole population. MGV: mean gray value, MG: medial gastrocnemius, SOL: soleus, SD: standard deviation. Significance level: *p* < 0.05.

	Hemiparetic Limb			Unaffected Limb		
	MG	SOL		MG	SOL	
	Mean (SD)	Mean (SD)	*p*-Value	Mean (SD)	Mean (SD)	*p*-Value
MGV	85.67 (21.87)	71.53 (15.93)	<0.0001	65.44 (18.54)	54.88 (14.82)	<0.0001

**Table 7:** MGV difference between ipsilateral MG and SOL on both affected and unaffected limb. Study population was stratified according to walking capability and level of spasticity. MGV: mean gray value, FAC: functional ambulation classification, MAS: modified Ashworth scale, MD: mean difference, SD: standard deviation. Significance level: *p* < 0.05.

	Hemiparetic Side		Unaffected Side	
	MGV MD (SD)	*p*-Value	MGV MD (SD)	*p*-Value
FAC				
≤3	14.731 (22.534)	0.0012	12.775 (17.958)	0.0005
>3	13.373 (21.583)	0.0070	7.661 (18.507)	0.0597
MAS				
≤2	11.108 (21.434)	0.0241	6.243 (20.080)	0.1595
>2	16.295 (22.364)	0.0003	13.616 (16.392)	<0.0001

## Data Availability

The data presented in this study are available on request from the corresponding author. The data are not publicly available due to privacy protection policy.
